# Five-year relative survival by stage of breast and colon cancers in northern Italy

**DOI:** 10.3389/fonc.2022.982461

**Published:** 2022-10-31

**Authors:** Lucia Mangone, Francesco Marinelli, Isabella Bisceglia, Maria Barbara Braghiroli, Angela Damato, Carmine Pinto

**Affiliations:** ^1^ Epidemiology Unit, Azienda Unità Sanitaria Locale-IRCCS di Reggio Emilia, Via Amendola 2, Reggio Emilia, Italy; ^2^ Medical Oncology Unit, Comprehensive Cancer Center, Azienda Unità Sanitaria Locale-IRCCS di Reggio Emilia, Viale Risorgimento 80, Reggio Emilia, Italy; ^3^ Department of Biotechnologies, University of Siena, Siena, Italy

**Keywords:** incidence, five-year survival, breast cancer, colorectal cancer, stage

## Abstract

The aim of this study is to present the 5-year relative survival by stage of breast and colorectal cancer patients in a northern Italian province. For the period 2013-2015, cases were selected from the Reggio Emilia Cancer Registry. Breast cancer patients were divided into 3 age groups: <45, 45-74 (the target screening population) and 74+. Colorectal cancers patients were classified into <50, 50-69 (the target screening population), and over 69 years. Carcinomas *in situ* and unknown stage were both excluded from the survival analyses. The five-year relative survival was estimated using the Pohar Perme method. During the period examined, 1,450 breast cancers and 992 colorectal cancer cases were registered. Analyzing in detail the patients with breast cancer for the entire 2013-2015 period, we noted that 50.4% were in stage I, 33.6% in stage II, 10.8% in stage III and 3.8% in stage IV. The stage was unknown in only 1.3% of patients (19 cases). The stage data of patients with colorectal cancer showed 24.5% were in stage I, 26.1% in stage II, 23.4% in stage III, and 24.6% in stage IV, and 1.4% unknown. Breast cancer 5-year survival was 100%, 89.7%, 71.4%, and 29.1% for stages I, II, III and IV, respectively and for colon cancer 96.7%, 83.4%, 70.8% and 16.2%, respectively.The presence of cancer screening, associated with effective treatments, account for the high survival rate of early-stage breast and colon cancers.

## Introduction

In 2020, about 55,000 new cases of female breast cancer and over 43,700 colorectal cancers (23,400 in men and 20,300 in women) were detected in Italy ^1.^Excluding skin cancers, breast cancer was the most frequently diagnosed cancer in women (30%); colorectal cancer represented the third cancer in men (12%) and the second in women (11.2%) (1)

As regards the trend, the breast cancer incidence rate showed a slight increase in Italy (+0.3%), especially in women aged 45-49 and 70-74 years (due to the extension of the screening program’s target population) as well as increased incidence in some regions of the South. The 5-year relative survival rate of women with breast cancer diagnosed in Italy in the period 2010-2014 was 87.8% overall (2), with no significant differences between younger women and older women, and survival decreases in older women. The 5-year relative survival of colorectal cancers diagnosed in the period 2005-2009 was similar in both sexes: 65.3% in men and 66.3% in women (2) and, also in this case, the values decreased with increasing age. The presence of screening has changed the physiognomy of breast cancer (3) and especially of colorectal tumour; adherence to colorectal screening made it possible to interrupt the adenoma-carcinoma sequence with a sharp decline in incidence (4). Furthermore, the availability of increasingly effective drugs and personalized therapies has made it possible to have a better impact also on metastatic tumor, with progress at least on mortality (5, 6). The incidence of cancer, previously the prerogative mainly of developed countries, is changing in the world with increases in cases also in developing countries (7) where early forms are increasing, albeit less and less than in developed countries (8). Survival for breast cancer is heavily influenced by the stage of the disease (9, 10) and by socio-economic conditions (11, 12). Significant improvements have been highlighted especially in the age groups that are included in mammography screening (13). In Italy there is a lack of recent data related to survival by stage therefore most of the data reported refer to American and older series data (14–16). A recent study on breast and colorectal cancer survival by stage showed that the impact of mammography screening can increase the breast cancer survival rate, while with colorectal screening the incidence is reduced (17).

The aim of this work was to present changes in stage distribution and 5-year relative survival of breast and colorectal cancer based on recent population data in a northern Italian province.

### Materials and methods

For the period 2013-2015, 1,450 breast cancers and 992 colorectal cancers in the Province of Reggio Emilia were selected, with follow-up on 31/12/2020. The main information sources of the Reggio Emilia Cancer Registry (RE-CR) are anatomic pathology reports, hospital discharge records and mortality data integrated with laboratory tests, diagnostic reports, and information from general practitioners. The RE-CR covers a population of 531,891 inhabitants and is considered a high-quality CR with a high percentage of microscopic confirmations (98.8% for breast cancer and 93.4% for colon) and a low percentage of DCOs (Death Certificate Only), below 0.1%) (18).

It is also one of the few Italian CRs to have data updated to 2020 already published (19). In the present study, breast cancer was divided into 3 age groups: <45, 45-74 years (target screening population) and over 74 years. Colorectal cancers were classified into 3 age groups: <50, 50-69 years (target screening population) and over 69 years. For breast and colorectal cancer, we performed the nonparametric test for trend for the difference in stage between years. Survival was analyzed in terms of stage.

The stage at diagnosis was calculated following the 8th edition of the TNM (Tumor, Node, Metastasis) (20). Carcinomas *in situ* (23 breast and 53 colon) and those with unknown stage (19 breast and 14 colon) are reported in **Table 2** but were excluded from survival analyses to permit a comparison with other studies. The five-year relative survival was estimated using the Pohar Perme method (21). We calculated the 5-year survival of cancers registered in the period 2013-2015. Relative survival, calculated for both cancer sites, is an estimate of net survival representing cancer survival in the absence of other causes of death, defined as the ratio of the proportion of observed survivors in a cohort of cancer patients to the proportion of expected survivors in a comparable set of cancer-free individuals. In this study we report 95% confidence intervals (CI) and we defined as statistically significant a p-value <0.05.

## Results

Among breast cancer patients, 67.1% were in the screening age group, 22.3% were over the age of 75, and the remaining 10.6% below age 50. The distribution of patients by year of diagnosis was similar over the years under study, with few fluctuations per period (33.6% in 2013, 31% in 2014 and 35.4% in 2015) (**Table 1**). In patients with colorectal cancer, there was a prevalence of males (54.3%) and older patients. Compared to breast cancer patients, colorectal cancer patients increased in the last year considered, from around 31.1% in the year 2013 to 34.7% in 2015 (**Table 1**).

**Table 1:** Patience characteristics, province of Reggio Emilia, 2013-2015.

Breast (C50)	n	%
**Overall** (Female)	1,450	
**Age group, y**		
<45	154	10.6
45-74	973	67.1
75+	323	22.3
**Years of diagnosis**		
2013	487	33.6
2014	449	31
2015	514	35.4
**Colorectum (C18-C20)**	n	%
**Overall**	992	
**Sex**		
Male	539	54.3
Female	453	45.7
**Age group, y**		
<50	65	6.5
50-69	322	32.5
70+	605	61
**Years of diagnosis**		
2013	309	31.1
2014	339	34.2
2015	344	34.7

### Breast cancer

Analyzing in detail the patients with breast cancer for the entire 2013-2015 period, we noted that 50.4% were in stage I, 33.6% in stage II, 10.8% in stage III and 3.8% in stage IV. The stage was unknown in only 1.3% of patients (19 cases). Looking at individual years, there was a stability in stage I (from 52.4% in 2013, 49.0% in 2014, and 49.8% in 2015), a slight decrease for stage III and finally a slightly increase in stage IV (from 3.1% in 2013, to 3.8% in 2014 and 4.7% in 2015), while in slight decline for unknown forms (**Table 2**). Concerning 5-year survival (**Table 3**), breast cancer presented a very high survival rate, overall equal to 88.9%, with statistically better results in the screening group (93.9%), than in young women (88.1%) and a decrease in older women (74.1%). Survival was 100% for stage I, 89.7% for stage II, 71.4% for stage III and 29.1% for stage IV, with significant differences.

**Table 2:** Stage of breast and colorectal cancer by year of diagnosis.

Breast (C50)	2013		2014		2015			Total	
	n	%	n	%	n	%	p-value	n	%
**Stage**									
I	255	52.4	220	49	256	49.8	0.42	731	50.4
II	151	31	154	34.3	182	35.4	0.14	487	33.6
III	58	11.9	52	11.6	47	9.1	0.16	157	10.8
IV	15	3.1	17	3.8	24	4.7	0.19	56	3.8
Unknown	8	1.6	6	1.3	5	1	0.35	19	1.3
**Total**	487	100	449	100	514	100		1,450	100
*in situ*	79	13.9*	75	14.3*	80	13.5*	0.81	234	13.9*
**Colorectum**	**2013**		**2014**		**2015**			**Total**	
**(C18-C20)**									
	**n**	**%**	**n**	**%**	**n**	**%**	**p-value**	**n**	**%**
**Stage**									
I	71	23	87	25.6	85	24.7	0.62	243	24.5
II	84	27.2	84	24.8	91	26.5	0.85	259	26.1
III	70	22.6	78	23	84	24.4	0.59	232	23.4
IV	83	26.9	82	24.2	79	23	0.25	244	24.6
Unknown	1	0.3	8	2.4	5	1.4	0.24	14	1.4
**Total**	309	100	339	100	344	100		992	100
*cin situ*	16	4.9*	8	2.3*	29	7.8*	<0.01	53	7.5*

*Calculated on the sum of the totals and the *in situ*:.

**Table 3:** Province of Reggio Emilia, 5-year relative survival overall, by stage and age.

Breast (C50), female	n	%	95% CI
Overall	1,450	88.9	85.3-94.2
**Age**			
<45	154	88.1	81.3-91.7
45-74	973	93.9	91.8-95.5
75+	323	74.1	63.4-82.1
**Stage**			
I	731	100	100-100
II	487	89.7	80.9-92.4
III	157	71.4	60.5-79.7
IV	56	29.1	16.9-43.3
**Colorectum (C18-C20)**	n	%	95% CI
**Overall**	992	66.7	62.0-70.9
**Sex**			
Males	539	67.2	60.8-72.8
Females	453	66.1	59.0-72.2
**Age**			
<50	65	71	58.1-80.5
50-69	322	78.7	73.4-83.2
70+	605	59.7	52.8-65.9
**Stage**			
I	243	96.7	68.8-99.4
II	259	83.4	72.2-90.4
III	232	70.8	60.8-78.7
IV	244	16.2	11.4-21.9

### Colorectal cancer

The stage data of patients with colorectal cancer referred to the same period 2013-2015. It showed 24.5% were in stage I, 26.1% in stage II, 23.4% in stage III and 24.6% in stage IV In the three years considered, there was a slight trend towards an increase in stage I and a not significant decline in stage IV(**Table 2**). Overall survival was 66.7%, higher in males and patients under 70 with values of 96.7%, 83.4%, 70.8% and 16.2% for stage I, II, III and IV, respectively (**Table 3**).

### Trends

**Figure 1:**
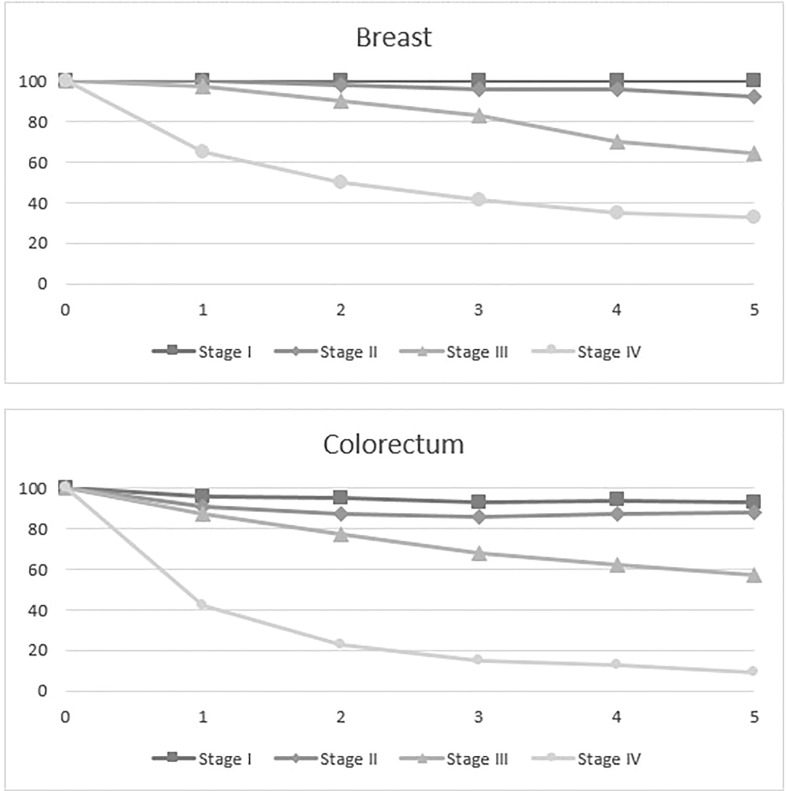
Province of Reggio Emilia, 5-year relative survival by stage and site.

**Figure 1** shows the trend of survival by year: breast cancer stages I, II and III show a similar decline (at 1 year above 97%, at 2 years above 93% at 3 years above 88% and 4 years above 78%) over the years, while stage IV decreases to 60% just 1 year from diagnosis. Also for colorectal cancer, stages I, II and III have a similar trend (> 90%, 87%, 79% 74%, respectively at 1, 2, 3 4 years after diagnosis), but the curve sharply decreases for stage IV to 42% in just one year after diagnosis. (**Figure 1**).

**Figure 2:**
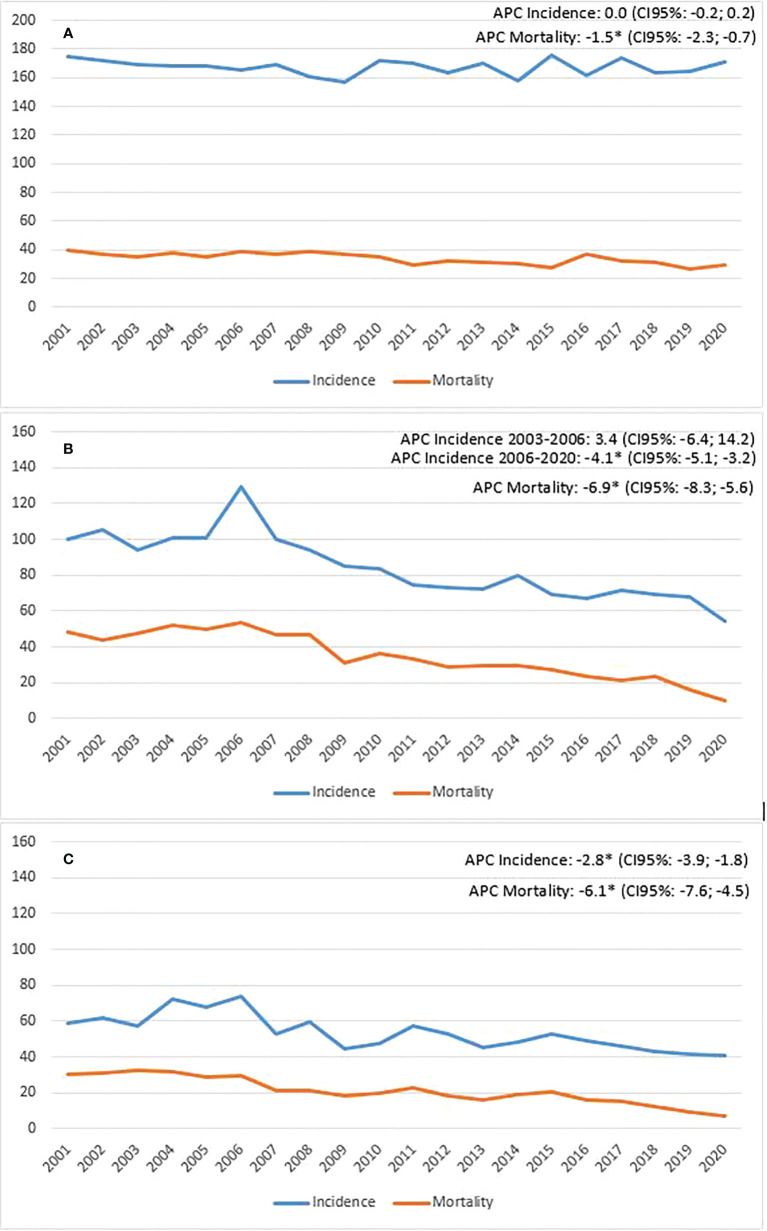
Province of Reggio Emilia, incidence and mortality trend (**A**=breast; **B**= colon, males; **C**=colon, females).

Finally, it is necessary to associate the changes seen in survival rates also considering what has happened in the distribution of incidence: in the 20 years observed, breast cancer has shown a slight and constant increase in incidence while colorectal cancer is in sharp decline in recent years (**Figure 2**).

## Discussion

The aim of this work was to show whether there have been changes in the distribution by stage of two major cancers, breast and colorectal, and the extent to which these changes have impacted survival rates. For breast cancer, stage I accounts for about half of all cancers diagnosed (50.4%), with no significant changes over the years. Alongside the stability in early-stage cancers, there is also the positive result related to the decline in advanced-stage cancers, which account for 3.8% of cases, compared to a national average of 6.4% (22). Since these are population data and not a selected case series, it means that screening, local health care situations, and the participation of general practitioners play an active role in raising public awareness of the importance of secondary prevention. Compared to the previous published paper (17), the distribution by stage shows no changes for stage I but shows a decrease in stage IV and unknown cancers. The latter data is important because it means that clinicians and CR registrars pay greater attention to recovering stage data of the disease. It is interesting to note that alongside the diagnostic anticipation, largely linked to oncological screening, advanced and metastatic forms have also decreased in our province, equal to 14.6% of cases, which is much less than what has been recorded in other places; for example, in Latin American countries they vary between 40% and 45% of cases (17). In our study, breast cancer 5-year survival was 88.9%, slightly down from the previous period 2010-2012 (91.4%), in particular linked to not significant decline in survival of stage III and stage IV (17). In a recent study, Siegel shows that for white women, 5-year survival was equal to 99% for the localized forms, 87% for the regional forms, 29% for the distant forms, while the overall survival was 91%, values comparable to those recorded in our study (100%, 78%, 32% and 91% respectively) (10).

Apart from stage, another determinant of survival is age. A Japanese study showed an improvement in 10-year survival for younger women (35-49 years) from 78% to 81%, and for women in the screening range (50-69 years) from 75% to 78% (13). In this current study, survival consistently remained about 90% up to age 74, and then dropped to 74.1% in older women. In Italy there are similar values: 91.4% in young women, over 93% in women aged 45-64, a slightly lower 91.7% in women aged 65-74, and 76.1%, in older women (75+) (2). Despite biological variables, the stage of the disease remains a strong determinant of prognosis for breast cancer. The risk of dying is 20 to 40 times higher in the large, high-grade, receptor-negative tumor group (9). Socioeconomic status is also one of the factors influencing survival (11). A Van der Meer study showed an improvement in survival for regional forms (from 85% to 94%) but only for women with high income, while for women with low income it remained at 84%. The same was shown for tumors with distant metastases (12).

Although part of the risk factors related to the development of these tumors are known and largely correlated to lifestyle and eating habits (23, 24), it would take a large number of subjects that for many years change the intake of fruits, vegetables and red processed meat to prevent one additional breast and colorectal cancer (25), naturally without forgetting the importance of genetic factors (26).

Among the many factors that come into play in the etiology of breast tumour, the role of hormone replacement therapy (27, 28) has been much debated over the years. Socio-economic factors (29) also come into play, which impact not only on incident but also on survival rates (30).

Reggio Emilia is a province in northern Italy characterized by high income, high-quality health care, and high adherence to cancer screening, factors which account for the high percentage of cancers registered in early stage. The highest survival rates (88.9%), compared with Northern Europe (81-84%), USA (84%) and Eastern European countries (69%), are linked to the fact that we have a high percentage of early cases (50.5%), higher than the US (39%) and European (32%) countries. This result is linked to the fact that Reggio Emilia has among the highest adhesions to mammography screening registered in Italy (31).

In the present study we considered only female breast cancers: male breast cancers, despite being a very low percentage of cases (1% of male cancers), are interesting when compared to female cancers both for their biological characteristics (32), familiarity (33) and stage (34).

As regards colorectal cancer, the most interesting data for 2013-2015 was the downward trend for stage IV over the years but the overall value (24.6%) remains similar to that registered previously. There is instead a decrease in stage unknown (1.4%) compared with 7.7% registered in 2010-2012 (17).Colorectal cancer showed a 5-year survival of 66.7%, with very high values for stage I and II but decreasing for stage III and dropping sharply for stage IV. It must be said that age is a strong determinant of survival in this case. While the values were higher in <50 (71.0%) and 50-69 (78.7%) age groups, survival dropped by almost 20% in elderly people (59.7%), which represent two-thirds of the cases. In fact, while the average age of our colon tumor patients is 72 years, this value rises to 75 years for M + tumors, which means that alongside the treatment of the tumor, other problems arise related to comorbidity and advanced age that limit its curability. Similar values by stage were reported by Siegel: survival of 90%, 72%, 15% and 65% for localized, regional, distant, and overall forms respectively (10). A Canadian study showed a wide regional variability, with stage I ranging from 92% to 98%, II from 88% to 95%, III from 83% to 88%, IV from 28% to 38%, however, data concerned only survival 2 years after diagnosis (35). Socio-economic factors also have an influence on colon cancer (36, 37): 5-year survival is 65% in high-income countries such as Australia, Canada, the U.S. and in several European countries, but less than 50% in low-income countries. Considering the colon and rectum separately, Innos reported survival for colon cancer equal to 90%, 86%, 71% and 15% for stages I, II, III, IV (38), similar to that recorded in Reggio Emilia (97%, 82%, 76% and 17%, respectively). For rectal cancer, survival was equal to 92%, 75%, 70% and 12% for stages I, II, III, IV, which seems similar, but stage III than that recorded in our data case studies (96%, 88%, 56% and13).

Among the strengths of this study we report that it is a population database: all registered breast and colorectal cancer patients are included in the study. This means that there are no selection biases that include only hospital-treated patients or patients included in cancer screening.

Therefore, a positive message for women should be to participate in cancer screening that ensures early diagnosis, and that this is associated with high survival. On the other hand, no significant improvement was seen in colorectal cancer survival, although a strong impact of screening was seen on incidence and mortality in both sexes and in any case the early forms have a better prognosis. Among the limitations of this study, there is the absence of biological variables which, especially in breast cancer, have a strong impact on survival. For metastatic cancers, the availability of innovative treatments may better explain some changes in survival rates.

## Conclusions

In our study, breast cancer survival was stable (although already high) in early stages. In fact, half of the breast cancers were stage I, with a 5-year survival of 100%, virtually as if these women had never had a tumor. Instead, a slight improvement in survival was seen for stages III and IV in the two age groups covered by the extension of screening (45-49 and 70-74 years). For colon cancer, while no significant improvement was seen by stage, screening had a strong impact on incidence and mortality; given the natural history of this tumor, screening resulted in interrupting the adenoma-carcinoma sequence. Of course, we cannot exclude that primary interventions have had a strong impact on the reduction of incidence, and that new treatments have contributed to reducing mortality.

## Data availability statement

The raw data supporting the conclusions of this article will be made available by the authors, without undue reservation.

## Author contributions

Conceptualization, investigation, writing—original draft, visualization, supervision, L.M.; formal analysis, F.M.; writing—review and editing, and visualization, I.B.; investigation, supervision MB.B.; investigation and supervision A.D; conceptualization, writing—original draft, investigation, and supervision, C.P. All authors have read and agreed to the published version of the manuscript

## Funding

This study was partially supported by Italian Ministry of Health - Ricerca Corrente Annual Program 2023.

## Conflict of interest

The authors declare that the research was conducted in the absence of any commercial or financial relationships that could be construed as a potential conflict of interest.

## Publisher’s note

All claims expressed in this article are solely those of the authors and do not necessarily represent those of their affiliated organizations, or those of the publisher, the editors and the reviewers. Any product that may be evaluated in this article, or claim that may be made by its manufacturer, is not guaranteed or endorsed by the publisher.
